# Development of a Caramel-Based Viscoelastic Reference Material for Cutting Tests at Different Rates

**DOI:** 10.3390/ma14143798

**Published:** 2021-07-07

**Authors:** Dennis Schab, Susann Zahn, Harald Rohm

**Affiliations:** Chair of Food Engineering, Institute of Natural Materials Technology, Technische Universität Dresden, 01062 Dresden, Germany; susann.zahn@tu-dresden.de (S.Z.); harald.rohm@tu-dresden.de (H.R.)

**Keywords:** cutting, ductile-brittle-transition, fracture, viscoelasticity, moisture, solid fat content, cutting speed, caramel

## Abstract

Cutting speed plays a crucial role for the behavior during and the final quality of viscoelastic foods after cutting and is, in industrial applications, usually adjusted on an empirical basis. Although previous studies investigated the interplay between the time-dependent properties and cutting behavior of model systems on an elastomer basis, there is still a need to elaborate such cause-effect relations for real foods. The aim of this study was to establish a reproducible manufacture of model caramels on a laboratory scale and to investigate the influence of the compositional parameters, moisture, and solid fat content, as well as cutting speed, on cutting behavior. It was possible to visualize ductile-brittle transitions in cutting force profiles, with an increase in cutting speed resulting in effects similar to that induced by a decreasing moisture content or an increasing solid fat content. Quantitatively, the progression of both maximum force and cutting energy reversed when cutting speed increased and composition changed in favor of a more brittle behavior. This work provides the basis for further research on distinct loading phenomena observed during the cutting of foods and for numerical modeling of the cutting process.

## 1. Introduction

Cutting is an important process in food production, aiming at separating commodities into pieces with defined macroscopic dimensions. This process is determined by the interplay between product properties and technical parameters related to the applied movement and the cutting tool [1,2,3,4]. When a soft material is penetrated, tensile stresses dominate in the deformation zone in the front of the blade. Once fracture tension is exceeded because of the deformation introduced by the blade, crack initiation and crack growth are responsible for the macroscopic separation of the material [5,6,7]. Friction forces between the blade and the newly formed cutting surface finally lead to shear stresses and lateral deformation. As a consequence, the cutting behavior of a particular product and the appearance of the resulting cut sections are strongly linked to its rheological properties.

The food industry usually processes agricultural materials into more or less complex products with a liquid, semi-solid, hard, or tough texture. In practice, foods can assume any condition between pure elastic and pure viscous behavior, and, in most cases, a time and deformation-dependent viscoelastic behavior is observed. As time dependency is responsible for phenomena such as stress relaxation and creep, the success of any cutting process largely depends on cutting speed. In the case it is low, i.e., when the kinetics of relaxation and creep processes in front of the cutting edge are faster than the chosen cutting speed, zones with extensive product deformation and irreversible structural damage must be expected in the vicinity of the blade. To prevent such effects during cutting, the apparent stiffness of the material can be increased by applying a higher cutting speed for reducing viscous energy dissipation or by separating at a lower temperature to reduce structural mobility and flexibility, thus taking advantage of the temperature-time superposition principle [8,9]. On the other hand, a high cutting speed leads to cleavage fracture events that cause imperfect separation planes and splintering of the product and, finally, may disrupt production [10,11]. In such situations, for instance, when strands of extruded goods are separated or when sausages or cheese are sliced, the time-dependent response of the material to the energy introduced by the cutting tool is of utmost importance [12]. Schuldt et al. [10,11] demonstrated the resulting effects by using a high-speed cutting station equipped with a 100 kfps camera. Apart from selected real foods, an elastomer filled with either solid particles (starch) or oil droplets was used in these experiments, and the mechanical properties of this elastomer served as a basis for the numerical modeling of crack propagation during cutting [13]. The fact is, however, that such synthetic models reflect foods by means of neither composition nor microstructure, and the challenge is to identify food systems with constant properties that exhibit a time or rate-dependent brittle-ductile transition, which is necessary for modeling the respective effects on cutting behavior.

The motivation for this study was, therefore, to develop a food-based material that consists of some or all of the main food components (protein, carbohydrates, fat, water), that can be reproducibly prepared in a laboratory environment and that exhibits the mentioned brittle-ductile transition when separated at different cutting speed. One of the possible systems is some sort of cheese analog, processed from protein, fat, water, and emulsifying salts by intense mixing and heating. In such systems, the material response largely depends on moisture content and on the thermal properties of the incorporated fat (solid/liquid ratio at a defined temperature) [14,15,16]. The second system that was, after preliminary experiments, finally chosen for this work is caramel candy, consisting of different types of sugar, protein, fat, and water, which are also mixed during heating to obtain a supersaturated sugar solution [17]. In this case, the mechanical properties of the final product mainly depend on final product moisture, which can be adjusted by the target heating temperature.

Caramel is one of the most produced confectionery products worldwide and available in a wide range of consistencies. It is either consumed as stand-alone confectionery or is used as an ingredient in more complex foods [18,19]. Caramel is usually made by cooking a mixture of water, sucrose, glucose syrup, milk solids, fats, and emulsifiers to a final moisture content of 6–12%, which requires a temperature of up to 118–125 °C [18,20]. Caramel exhibits a complex microstructure comprised of a continuous amorphous sugar matrix with dispersed fat droplets, a protein network, and air bubbles [21,22]. In addition to the dominating effect of moisture, each of the bulk ingredients introduces specific functions into the system with respect to structure and mechanical behavior.

- Sucrose and glucose syrups are bulk ingredients and sweeteners in caramels as well as in fudge and toffee, giving the products their unique texture and flavor. The ratio of sucrose to glucose syrup, a starch hydrolysis product that also contains small amounts of maltose and larger oligosaccharides, determines the extent of sucrose crystallization during and after production. Glucose syrup prevents graining [23] by increasing bulk viscosity which, in turn, decreases the molecular mobility of sugar crystal seeds [19,24]. Higher levels of glucose syrup are necessary to obtain an ungrained and smooth texture, whereas excessively high levels make products sticky and prone to cold flow [25].
- The rationale behind adding dairy ingredients is to incorporate milk proteins, usually by adding sweetened condensed milk, evaporated milk, or milk powder [25]. Casein, the main milk protein, provides structure by forming a network throughout the sugar matrix upon heating and by stabilizing the fat droplets through the formation of layers on the sugar-fat interface [21]. The whey protein contributes to non-enzymatic browning with reducing sugars, including lactose, bringing up the typical caramel flavor and light brown color [22,26].
- Fat is the third major component that contributes to flavor and texture, gives the product a smooth mouthfeel by reducing stickiness generated by the sugar compounds [19], and provides lubrication for improved machinability, including cutting [27]. This is because fat droplets partly replace the sticky components, namely sugar, on the surface in contact with a tool [28]. As the fat droplets can be regarded as filler in a continuous polymeric matrix, the melting properties of a specific fat also contribute to caramel firmness and product defects such as cold flow [29].

This manuscript gives information concerning laboratory preparation and the mechanical properties of a stable, caramel-based viscoelastic material with a brittle-ductile transition at a cutting rate that can be adjusted by adapting processing conditions and formulation.

## 2. Materials and Methods

### 2.1. Materials

Granulated sucrose was obtained from Südzucker AG (Mannheim, Germany), 42 DE glucose syrup from Cargill Deutschland GmbH (Krefeld, Germany), and skim milk powder from Sachsenmilch Leppersdorf GmbH (Wachau, Germany). Palm oil (Bavettin 22820) and a refined vegetable fat powder (Palmetta 70560/SG) with a solid fat content (SFC) of 21% and 94% at 20 °C, and a slip melting point of 36 °C and 60 °C, respectively, were obtained from Walter Rau AG (Neuss, Germany). Sunflower lecithin served as an emulsifier and was supplied by Cargill s.r.l. (Padova, Italy).

### 2.2. Sample Preparation

After some preliminary experiments, the base formulation used in this study comprised 29.4% sucrose, 29.4% glucose syrup, 24.4% deionized water, 8.0% skim milk powder, 7.9% fat, and 0.9% lecithin. The moisture content of the final caramels was adjusted through the final temperature during cooking. The formulation further denoted as reference (Ref) contained both the palm oil and the vegetable fat powder in equal amounts. The alternative formulation SFC1 comprised solely of the soft palm oil, and formulation SFC2 only contained the refined hard fat.

All products were cooked in a stainless steel pot in 460 g batches. After dissolving the skim milk powder in water, all further ingredients were added step by step while mixing with an inclined-blade stirrer attached to a Eurostar 60 device (IKA-Werke GmbH & Co. KG, Staufen, Germany) at 250 rpm for approx. 1 min. The pot was then placed on a 2000 W induction heater (neoLab Migge GmbH, Heidelberg, Germany) set to 200 W. This power level was kept constant throughout the entire cooking procedure. The temperature of the caramel mass during cooking was continuously measured using a 175-T3 temperature logger equipped with a thermocouple type T needle probe, reaction time approx. 2 s (Testo SE & Co. KGaA, Titisee-Neustadt, Germany). This probe was mounted on a support stand in such a way that it reached vertically into the mass for about 30 mm, with its tip slightly above the bottom of the pot. During mixing up to 70 °C, the stirrer speed was set to 250 rpm. When all components were liquefied or dissolved, the rotational speed was increased to 700 rpm to emulsify the fat into fine droplets and to homogenize the mass. Once the mass reached 90 °C, the stirrer speed was set back to 250 rpm. From 100 °C onwards, stirring was superseded by the manual scraping of the bottom surface with a heat-proof plastic scraper to prevent scorching. At the target final temperature, which was between 114 °C and 120 °C, the pot was immediately removed from the induction heater and transferred into a bowl with crushed ice for about 10 s to remove the bulk heat. The caramel mass was then quickly poured into PTFE molds using plastic syringes with cut-off tips to obtain cylindrical specimens (*d*, 12 mm; *h*, 10 mm) for compression tests and cubical specimens (edge length, 20 mm) for cutting experiments. The molds were immediately packed in zip bags with a minimized headspace and stored at room temperature (22 ± 1 °C) until analysis.

### 2.3. Experiments on Properties Related to Cooking Temperature and Storage Stability

To obtain data on the reproducibility of the cooking procedure, all caramel formulations were cooked in quadruplicate to a final temperature of 120 °C. In two cases of each formulation, samples were taken during heating at 108 °C, 111 °C, 114 °C, 116 °C, 118 °C and 120 °C. Samples taken at 108 °C and 111 °C were filled into small sample cups as they did not solidify during cooling. For all other temperatures, the samples were filled into 20 × 20 × 10 mm^3^ PTFE molds, stored overnight at room temperature, and ground immediately before moisture content and water activity was analyzed in duplicate. The final batches were also molded into cylinders for subsequent stiffness testing.

For the evaluation of the storage stability of the caramels, another set of experiments was performed. Four batches of the reference caramel were cooked to a final temperature of 120 °C (*n* = 2) or 115 °C (*n* = 2), molded as described above, and subsequently stored at room temperature. Moisture content, water activity, the modulus, and thermal properties were analyzed 24 h after preparation and then in daily increments up to 4 days.

### 2.4. Analytical Methods

#### 2.4.1. Moisture Content

Vacuum drying was used for determining the moisture content of the caramels in duplicate. After removing the samples from the zip bags, they were grated using a cheese grater. Approx. 0.5 g was transferred into glass dishes with a lid. After weighing the samples, they were dried in a VT 6060 M vacuum chamber (Thermo Fisher Scientific, Waltham, MA, USA) at 60 °C and a pressure < 2 kPa for at least 64 h until mass constancy was reached. Moisture content was calculated gravimetrically as percentage loss on drying.

#### 2.4.2. Water Activity

Water activity was measured at room temperature using a LabMASTER-aW instrument (Novasina AG, Lachen, Switzerland). The grated samples were filled into 15 mL sample cups with a filling level of at least 2/3 of the height of the cups. The instrument was set to continuous reading mode, and water activity was taken when it was constant for at least 3 min. Unless stated otherwise, each caramel was tested in triplicate.

#### 2.4.3. Differential Scanning Calorimetry

Thermograms were recorded using a Discovery DSC25 differential scanning calorimeter connected to an RCS90 cooling unit (TA Instruments, New Castle, DE, USA). Nitrogen was used as purge gas at a flow rate of 50 mL/min. Calibration of the instrument was performed using indium and gallium as standards. The tests were carried out in the modulated mode, which allows separating the heat flow into a reversing and a non-reversing signal.

An amount of 8–10 mg sample was weighed into standard aluminum DSC pans that were then covered with a lid. An empty pan was used as a reference. In the cell, the samples were immediately cooled to −50 °C and equilibrated for 3 min. This was followed by heating to 35 °C with a rate of 2.5 K/min, and a superimposed modulation temperature and period of ±1 K and 60 s, respectively. All measurements were performed in duplicate.

An integrated analysis tool in the TRIOS software was used to determine the glass transition onset (*T_G,on_*, °C) and endpoint temperature (*T_G,end_*, °C) from the reversing heat flow signals. Additionally, the specific heat capacity at *T_G,on_* and *T_G,end_* was used to calculate the transition-induced change in specific heat capacity (Δ*c_p_*, J/g·K). For a schematic representation of how these parameters were determined, see Sritham and Gunasekaran [30].

#### 2.4.4. Compression Tests

An RSA3 Solids Analyzer (Rheometric Scientific Inc., Piscataway, NJ, USA) was used to conduct compression tests at small deformation using a parallel plate geometry. Cylindrical specimens were removed from the molds immediately before testing. After measuring specimen diameter using a digital caliper, they were placed on the lower plate and lubricated with paraffin oil. The upper plate was lowered until there was full contact with the surface, and the height of the individual specimens was read from the instrument’s display. Compression was carried out at a speed of 0.1 mm/s. Stiffness refers to Young’s modulus (*E*, Pa), calculated from the slope of normal stress vs. Cauchy strain in the linear region. All tests were performed in septuplicate at room temperature (22 ± 1 °C).

### 2.5. Cutting Experiments

Based on the method established by Schuldt et al. [6,7], cutting experiments were performed at room temperature using a 5564 universal testing machine (Instron Ltd., High Wycombe, UK). The instrument was equipped with a 1 kN force transducer that was fixed at the bottom of the load frame and that carried an aluminum sample support rig. A cutting blade made from electrically polished steel (70 × 20 × 1 mm^3^, wedge angle 20°, wedge height 3 mm, ASTOR Schneidwerkzeuge GmbH, Storkow, Germany) was fixed in a clamp that was mounted on the instrument’s crosshead. To obtain force and displacement data with a higher resolution as could be provided by the instrument software, analog output signals were recorded with an NI9215 data logger (National Instruments, Austin, TX, USA) with a sampling rate of 1 kHz and retransformed into force and deformation signals using the appropriate calibration data.

Immediately before measurement, the cubical specimens were placed on the sample support rig, and the blade was lowered until full contact with the sample surface was ensured. Force and displacement readings were set to zero, and the blade was raised by exactly 10 mm before the measurement started. The applied cutting speed was 400, 1000, or 2500 mm/min, and the specimens were cut to a depth of 15 mm. This means that, at the highest cutting speed, force and displacement were read at a sampling rate of approx. 25 data points per mm. All tests were performed at room temperature (22 ± 1 °C) in quadruplicate, and the blade was wiped with ethanol after each measurement. The resulting force-displacement time data triplets were evaluated with respect to maximum cutting force (*F_max_*, N) and cutting energy (*W_C_*, J), referring to the area under the force-displacement curve.

### 2.6. Statistics

All data are expressed as mean ± standard deviation (*n* > 2) or mean ± half deviation range (*n* = 2). Data were statistically analyzed by a regression analysis and univariate ANOVA with Tukey’s post hoc test for multiple means comparison using Origin 2019b (OriginLab Corporation, Northampton, MA, USA).

## 3. Results and Discussion

### 3.1. Impact of Cooking Temperature on Caramel Moisture and Stiffness

**Figure 1:**
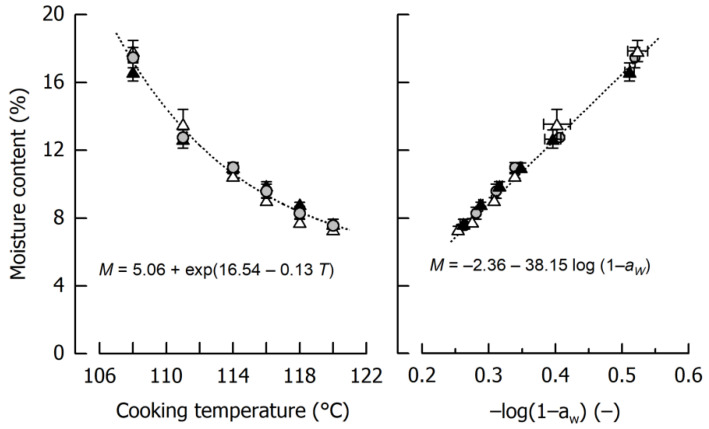
Dependency of caramel moisture content *M* on cooking temperature *T* (**left**) and relation between moisture content and water activity *a_W_* (**right**). Data are arithmetic mean ± standard deviation from 2 independent preparations per formulation and duplicate measurements (*n* = 4). Symbols: Formulations Ref (grey circles), SFC1 (black triangles), SFC2 (open triangles). Equations refer to the fits indicated by dotted lines.

Figure 1 shows the impact of cooking temperature on the moisture content of the different caramels. In the considered temperature range, product moisture ranged between 17.31 ± 0.53% after cooking to 108 °C and 7.52 ± 0.17% after cooking to 120 °C. Using the entire data set, caramel moisture can be expressed as an exponential decay function of cooking temperature (*r* = 0.99, *p* < 0.01). It is also evident that the influence of the type of fat incorporated in the formulation is negligible, as the slip melting point of the harder fat is below the temperature where emulsification started. From this point of view, it can be expected that the oil droplets in the final caramels showed a similar size distribution [20].

Another important observation from the experiments displayed in Figure 1 is that water activity *a_W_* was, after logarithmic transformation [31], linearly related to caramel moisture (*r* = 0.99, *p* < 0.01). In addition to conceptual differences—it is mainly free water that contributes to *a_W_*—this logarithmic transformation is based on the fact that the absolute amount of solids dissolved in the continuous phase does not linearly increase with increasing moisture. The observed dependency implies that when a particular moisture content of the caramel is aspired, it can be indirectly judged and classified by measuring water activity. This is important because *a_W_* results can be achieved in a time scale comparable to Karl Fischer titration (which is not available in each laboratory) but approx. two magnitudes of time faster than necessary to obtain mass constancy when using the oven method.

**Figure 2:**
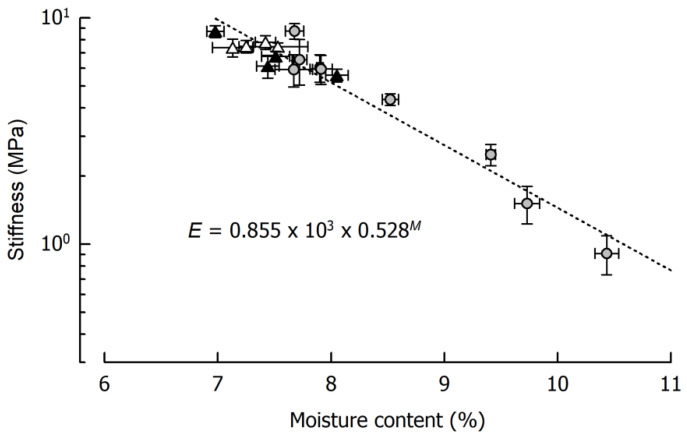
Relationship between caramel stiffness *E* and moisture content *M*. Data are arithmetic means ± standard deviation for stiffness (*n* = 7) and for moisture (*n* = 3). Symbols: Formulations Ref (grey circles), SFC1 (black triangles), SFC2 (open triangles). Equation refers to the fit indicated by the dotted line.

Figure 2 displays caramel stiffness and the dependency on its moisture content for the three formulations that were cooked to 120 °C in quadruplicate and, in addition, for the two samples cooked to 116 °C and 118 °C as well as for the samples cooked to 115 °C or 120 °C for the storage experiments. It is evident that, in the case of a final cooking temperature of 120 °C, a caramel moisture of 7–8% could be achieved (see also Figure 1). It is also evident that such relatively small deviations in moisture resulted in pronounced differences in sample stiffness, ranging from 5.9 MPa to 8.7 MPa. In some of the samples, the between-measurement deviations were relatively large, presumably because of specimen inhomogeneity or inaccuracies in sample geometry that could not be entirely avoided. It can further be seen from Figure 2 that, at least as concerns small deformation stiffness, the solid fat content of the caramels played a minor role. There was a tendency that, at comparable moisture, the caramels containing the soft fat were slightly softer. This effect is, however, superimposed by the pronounced influence of moisture when samples cooked to lower temperatures are taken into account (*r* = −0.97, *p* < 0.01).

### 3.2. Storage Effects and Reproducibility of the Caramel Model Systems

Depending on available moisture and the solubility of sucrose in the presence of glucose and lactose, amorphous sucrose in caramels tends to recrystallize to a certain extent during storage [32,33]. In turn, the crystal content has a significant impact on the mechanical properties of caramels [34]. As a consequence, the reproducibility of cutting tests can only be ensured with samples being considered stable. It was, therefore, necessary to identify the time frame in which changes of the samples are negligible. Table 1 shows water activity, thermal behavior at the glass transition, and the stiffness of four batches of the reference caramels that were measured in daily intervals for up to 4 days. The experiments were carried out solely for the reference formulation since the fat in caramels is dispersed and non-interacting [34] and, therefore, not expected to influence sugar crystallization.

Water activity connects to crystallinity in that a higher amount of crystalline sucrose leads to reduced interaction with water as the amount of sucrose dissolved in the continuous phase becomes lower [20,34]. Regarding sugar-based foods, this was also reported in the case of, for instance, honey [35,36]. For the batches cooked to 120 °C, water activity slightly increased over four days, presumably because of a higher amount of free water in the continuous phase induced by some sucrose crystallization. Generally, however, *a_W_* changes along the observed time frame were largely insignificant (*p* > 0.05), pointing to only minor changes in crystallinity. When interpreting significances in the *a_W_* data set, the high sensitivity of the measuring instrument, indicated by the rather low standard deviations, should also be considered.

It is also sucrose crystallinity that affects the mechanical properties of caramels. An increase in the crystalline fraction means that sucrose molecules become excluded from the continuous phase but are instead present as stiff filler particles dispersed in the matrix, therefore significantly contributing to stiffness [7,34]. In line with the findings from *a_W_* measurements, caramel stiffness did not change significantly over four days (*p* > 0.05), except for the caramel mass containing approx. 9.7% moisture. We hypothesize that this observation can be explained by structural mobility effects that influence crystallization dynamics. As pointed out by Hartel and Shastry [37], crystal growth rate increases with decreasing moisture and increasing supersaturation up to a point where structural mobility limits diffusion, so that crystal growth is inhibited. In low-moisture or glassy systems, on the other hand, crystallization might be induced by the plasticizing effect caused by an increasing moisture content [38,39]. Thus, sample Ref_115C_1 might have contained a suitable amount of moisture for crystallization, considering rate and amount, to become detectable in compression tests in the observed time frame. A similar tendency, although more subtle and without being significant, is evident for sample Ref_115C_2.

Differential scanning calorimetry is a well-established method to evaluate amorphous and crystalline fractions of carbohydrates [40,41,42]. While it is possible to estimate the amorphous fraction from crystallization and melting enthalpy [40,43], another approach probably more appropriate for most multi-component foods is to investigate glass transition. When doing so, the amorphous fraction can be indirectly quantified from the change in specific heat capacity that is induced by glass transition. This parameter relates to the degree of organization and the level of hydrogen bonding in a glassy matrix [44,45], it is associated with changes in structural mobility [46], and it is linearly related to the amorphous sugar content [41,42,47]. We used this proportionality to indirectly verify whether there were any significant changes in the ratio of amorphous and crystalline fractions in the caramels without necessarily having to calculate the precise values of these fractions (which would have required some sort of reference material). By using the reversing signal from modulated DSC, thermal processes that result in a change in heat capacity such as glass transition or melting can be analyzed separately from any other potentially overlapping processes such as enthalpy recovery [30].

From the DSC results (see Table 1), it is evident that the glass transition of the caramels takes place close to the freezing point of water. The span from glass transition onset to endpoint comprised approx. 12–15 K. Similar temperature ranges for caramel systems were reported by Ahmed et al. [18], although the absolute *T_G_* reported in that study was considerably lower, presumably because of a higher moisture content and other differences in the formulation. The plasticizing effect of water is reflected by a lower *T_G_* [30,38,48], which is also evident from our results when comparing the samples with different moisture content. For the caramels cooked to 120 °C, showing 7.68% and 7.64% moisture, both onset and endpoint temperatures were nearly identical. It was interesting to observe that the width of the glass transition region slightly broadened with increasing moisture content. This is in contrast to the findings of Sritham and Gunasekaran [30] and Borde et al. [46], who reported a decreasing glass transition width with increasing moisture for sucrose and amylopectin, respectively. However, the increase in our results was relatively small (~2 K), and differences in measuring parameters, for instance, heating rate, and composition have to be taken into account [49]. On the other hand, our results are in line with a report on plasticizing additives that broaden the glass transition region of synthetic polymers [50].

In total, Δ*c_p_* ranged from 0.42–0.52 J/g·K among the caramel samples which is slightly lower than data on sucrose (0.54–0.65 J/g·K, [51,52,53,54]) and comparable to data on maltodextrin (0.44–0.45 J/g·K, DE19–DE20, [44,55]. This is plausible since the caramels are, to a large extent, a mixture of sucrose and polymerized glucose. Δ*c_p_* also increased with increasing moisture, pointing to an increased change in molecular mobility caused by the water molecules. No significant changes in Δ*c_p_* were found for any of the samples during storage that could be associated with sucrose crystallization, generally confirming the results of the other methods. Nevertheless, the slight but insignificant decrease in the mean values from day one to day four may be taken as an indicator for the presence of some crystallization effects, similar to the *a_W_* results. Since glass transition temperatures, Δ*c_p_* and *a_W_*, are matching for the caramels cooked to 120 °C, it can be concluded that repeated cooking to the same moisture leads to comparable caramel properties and, hence, to the assumption that caramel moisture is a suitable criterion to assure the reproducibility of caramel cooking. Based on these results, subsequent cutting tests were chosen to be conducted within two days after sample preparation.

### 3.3. Effects of Caramel Moisture on Cutting Behavior at Different Cutting Speeds

**Figure 3:**
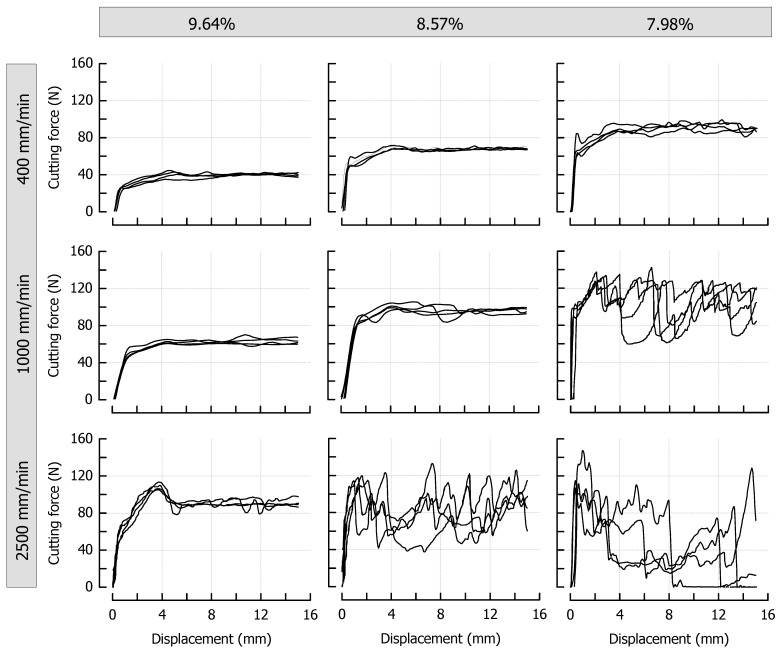
Impact of caramel moisture and cutting speed on cutting force in the reference formulation (*n* = 4). The moisture contents are denoted as percentage (*w*/*w*) above the corresponding diagrams.

Figure 3 shows the force profiles obtained at different cutting speeds for the reference caramels that were cooked to a final moisture content of 9.64%, 8.57%, or 7.98%. From a qualitative point of view, the effects of cutting speed and moisture content are similar: both an increase in cutting speed at identical moisture and a decrease in moisture at identical cutting speed result in an increased cutting force. Concerning cutting speed, the observations are in line with the results of previous cutting experiments using a viscoelastic model system on an elastomer basis [7,13] or real foods [10,11]. The shortened time scale for microstructural rearrangement processes that depend on the extent of viscous contributions in a material has already been discussed in this context [7]. In our current experiments, the decrease in caramel moisture led to the same observation. This is most probably due to reduced molecular mobility, which means that relaxation times are longer at any given deformation rate [56]. After achieving a particular deformation level, force-displacement curves obtained during the cutting of foods exhibit a steady-state cutting force plateau when the contributions from deformation, fracture, and friction are in equilibrium [5]. In the case of the caramels, this plateau was reached at a cutting depth of approx. 5 mm for all samples that do not show a pronounced brittle crack propagation. The lack of an increase in cutting force with an increasing contact area between blade and product [1,57] points to considerably low friction forces at the blade-sample interface, presumably caused by lubricating effects of fat and moisture. Moreover, there was some additional lateral compliance of the samples at both sides of the blade due to the enhanced plasticity, especially at a higher moisture content. For a demonstration of the cutting experiments, please refer to the supplementary videos related to this article.

Another observation that is clearly evident from Figure 3 is the occurrence of brittle crack propagation and subsequent fracture, which happens when the dissipated viscous energy falls below a certain level, and the energy that is necessary for fracture is exceeded [12]. This transition from ductile to brittle usually cannot be analyzed by conventional methods [10,11] but can either be judged by the degree of jaggedness of the force curves or the point at which there is a sudden and permanent drop to zero force (full separation of the sample). The transformation of the force profiles from steady state (Video S1) into an increasingly brittle behavior (Videos S2 and S3) is evident for both an increasing cutting speed at a particular moisture, but also for a decreasing moisture at a particular cutting speed. A jagged force response implies that the blade tip is periodically losing contact with the material each time a new crack is initiated (see Figure 3, high speed/medium moisture or medium speed/low moisture, see also Video S2). It is further possible that the caramel with the lowest moisture that was cut at the lowest speed (Figure 3, upper right, see also Video S1) and the caramel with the highest moisture that was cut at the highest speed (Figure 3, lower left) already exhibit a slightly brittle behavior with crack propagation on a microscopic scale. This is indicated by the more or less jittery, unsteady progression of the force profiles. When certain factors such as higher speed or lower moisture lead to higher stresses in front of the blade, more energy is released by the formation of longer cracks, which finally, results in immediate and pronounced drops of the cutting force (see Figure 3, lower right). The low reproducibility, which is obvious for curves where brittleness is more important, comes from the random nature of crack formation that is related to the inner structure of a material and its inhomogeneity [58,59,60]. Anisotropy or small voids and inclusions may cause a somewhat undirected formation of crack paths, including a noticeable lateral deviation from the cutting plane. This is reflected by the recurrence of cutting force increases from about 8 mm blade displacement onwards. At this point, the blade penetrated the material again after there were considerable anticipatory cracks almost to the bottom in most of the samples (see Figure 3, lower right, see also Video S3).

**Figure 4:**
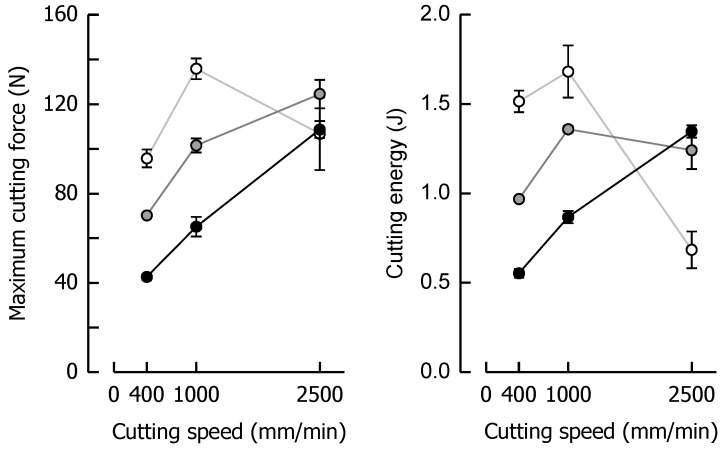
Relationships between maximum cutting force and cutting speed (**left**) and cutting energy and cutting speed (**right**) for caramels with 9.64% (black), 8.57% (grey), and 7.98% (white) moisture content. Data are arithmetic means ± standard deviation for maximum cutting force or cutting energy (*n* = 4) and for moisture (*n* = 3).

Maximum cutting force *F_max_* and cutting energy *W_C_* that were chosen for the quantitative analysis of the data set (same samples as in Figure 3) are depicted in Figure 4, giving a clear indication of the progress of these parameters with an increasing cutting speed when moisture content changes. For example, when caramel moisture increased, both *F_max_* and *W_C_* decreased at low and medium cutting speeds. This tendency, however, reverted at a cutting speed of 2500 mm/min. This seems reasonable for *W_C_* as crack growth tends to eliminate the fracture energy portion of the total energy. In the context of *F_max_*, a decrease in fracture toughness with brittle fracture as well as a decline in viscous energy dissipation because of reduced plastic deformations have been discussed [60,61]. Theoretically, when cutting speed increases but cutting force decreases, the energy input rate (i.e., power = energy/time = force × cutting speed) remains almost constant. This, in turn, means that less energy has to be used when the time scale becomes shorter (lower fracture toughness). For the samples with the highest moisture content, where there was no or only marginal crack growth during cutting, the data clearly point to a linear dependency of both parameters on cutting speed (*r* ≥ 0.99, *p* < 0.05).

**Figure 5:**
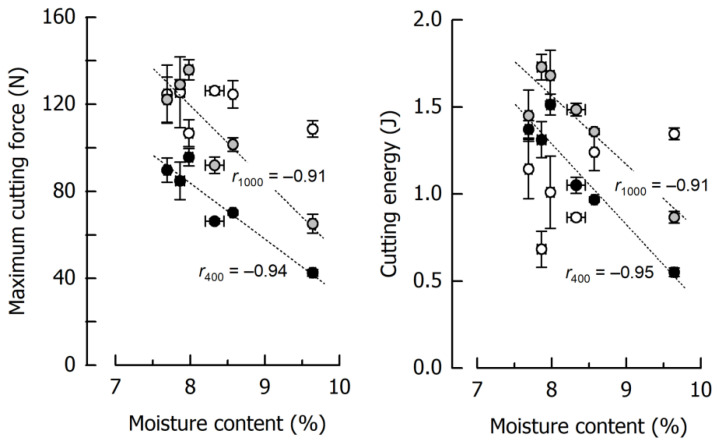
Correlations of maximum cutting force and cutting energy with moisture content at 400 mm/min (black), 1000 mm/min (grey), and 2500 mm/min (white) for the caramel reference formulation. Data are arithmetic means ± standard deviation for maximum cutting force or cutting energy (*n* = 4) and for moisture (*n* = 3).

The results for *F_max_* and *W_C_* of six reference caramels individually cooked to final temperatures of 117.5–121.4 °C, thus giving moisture contents ranging between 9.64% and 7.69% (including the samples from Figure 3), are depicted in Figure 5 as a function of the caramel moisture. There is a significant inverse dependency of *F_max_* and *W_C_* on moisture content at low and medium cutting speeds (*r* ≤ −0.91, *p* < 0.01). However, this is not true for a cutting speed of 2500 mm/min where it was more difficult to obtain reproducible data, especially for *W_C_*. The lower-than-expected values with high variability obtained for the low moisture samples, caused by the rather unpredictable crack formation, were accompanied by increasing fracture energy when moisture content increased. High-moisture caramels cut at 2500 mm/min were more ductile and tough and, hence, resisted fracture [62,63].

Figure 5 further illustrates the remarkable effects of caramel moisture on cutting force and cutting energy: an increase in moisture content from 7.67% to 9.64% decreased *F_max_* by 56% and 52% at 400 and 1000 mm/min cutting speed, respectively. The more pronounced slope of the regression line for 1000 mm/min points to an increasing dependence of the cutting force on the moisture content when cutting speed is elevated. For the same increase in moisture, *W_C_* was reduced by 60% and 50% at 400 and 1000 mm/min, respectively. The reduction in both *F_max_* and *W_C_* can be attributed to a significant reduction in deformation energy due to a higher structural mobility caused by the water molecules [56,61]. In the case of a cutting speed of 2500 mm/min, the regression coefficient between either *F_max_* or *W_C_* as a dependent variable and moisture content as an independent factor was below significance, and the respective trendlines are therefore not included in Figure 5.

### 3.4. Effects of Solid Fat Content on Cutting Behavior at Different Cutting Speeds

**Figure 6:**
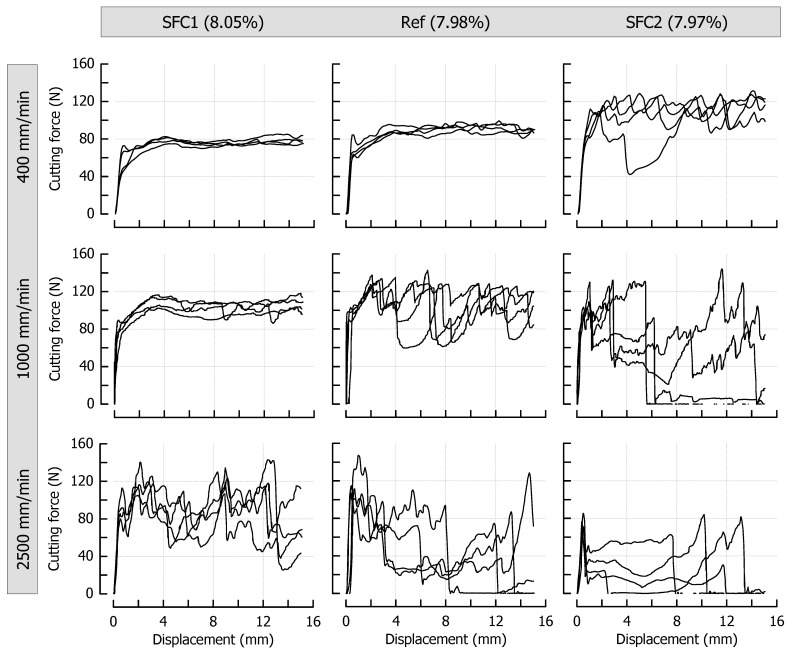
Impact of solid fat content and cutting speed on cutting force (*n* = 4). The moisture contents are denoted as percentage (*w*/*w*) above the corresponding diagrams.

Figure 6 shows the cutting force profiles for the formulations SFC1 (only soft fat), Ref (both fats in equal amount), and SFC2 (only hard fat), each cooked to a similar final moisture content of approx. 8% (*p* > 0.05), and therefore introduces solid fat content (SFC) as a third variable having a significant impact on the ductile-brittle transition of the caramels. The SFCs of these samples at 20 °C were, based on the fat fraction, 21% (soft fat), 57.5% (fat mixture), and 94% (hard fat), respectively. To allow a direct comparison, the reference caramel with 7.98% moisture is the same as the one that is shown in Figure 3. Similar to the effect of decreasing moisture, an increase in the solid fat content caused the samples to behave more brittle, which is again reflected by the more pronounced jaggedness of the force profiles. When the fat molecules start to build a crystal network within the dispersed fat globules, they become stiffer, thus acting as increasingly solid filler particles in the continuous phase. This leads to a decrease in the overall deformability of the matrix [7,20]. While the distinct SFC levels could not be sufficiently discriminated in small deformation compression tests, this effect was now evident in the large deformation cutting tests where tensile stresses in front of the blade tip become an important component of the whole loading process [13]. The increase in cutting speed also added up to the current level of brittleness at a given SFC. A marked qualitative similarity in the progression of the cutting force can be noticed when comparing diagrams that are diagonally opposite (from lower left to upper right, for instance, SFC1 at 1000 mm/min and Ref at 400 mm/min). In these particular cases, the effects of speed and SFC levels on cutting force compensate each other. This is also congruent with the force results obtained during cutting of caramels with different moisture content (see Figure 3), where moisture and speed levels are interchangeable in the same manner (for instance, when comparing caramels with 9.64% moisture cut at 1000 mm/min and caramels with 8.57% moisture cut at 400 mm/min).

**Figure 7:**
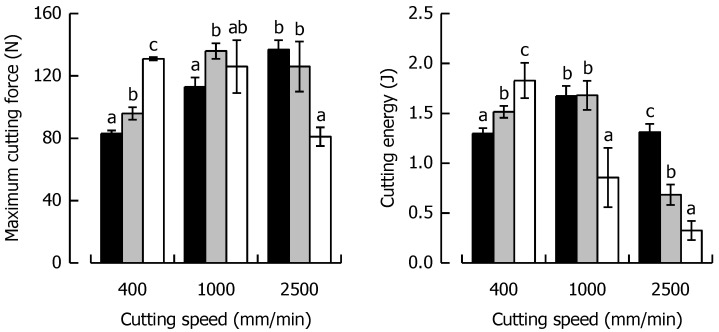
Relationship between maximum cutting force and cutting speed (**left**) and cutting energy and cutting speed (**right**) for the caramels with different solid fat contents. Colors: Formulation SFC1 (black), Ref (grey) and SFC2 (white). Data are arithmetic means ± standard deviation (*n* = 4). Different letters in a block indicate significant differences (*p* < 0.05).

*F_max_* and *W_C_* at the respective cutting speed for the caramels with different solid fat content are shown in Figure 7, indicating a similar behavior for both parameters, as was evident for the caramels with different moisture. *F_max_* at 400 mm/min increased by 60% when SFC increased from a low (21%) to a high level (94%). On the contrary, at 2500 mm/min, *F_max_* decreased by 41% for the same change in SFC because of the shortened time scale for viscous energy dissipation, responsible for lower fracture toughness and increased brittleness.

When SFC remained constant but cutting speed increased by a factor of 6.25 from low to high, there was a 67% increase and a 38% decrease in *F_max_* for SFC1 and SFC2, respectively. In the context of *W_C_*, there was a 41% increase at 400 mm/min when SFC increased from the low to the high level. The data suggest that this trend is significantly linear (*r* = 0.99, *p* < 0.05). At 2500 mm/min, a drastic decline in *W_C_* by 75% was found for the same change in SFC. Besides the already discussed reduction in viscous energy dissipation causing brittleness, it has to be considered that a reduced contribution of friction might have played a role in this decline of *W_C_* (and also *F_max_*). There is evidence that a higher SFC causes enhanced lubrication in emulsion-filled food gels [64]. The SFC1 caramel already showed a reversing behavior, with a significant increase in *W_C_* when cutting speed was elevated from 400 to 1000 mm/min (*p* < 0.05) but a return to the same level when speed was further increased to 2500 mm/min (*p* > 0.05). *W_C_* was most drastically reduced (82%) for the SFC2 caramel when cutting speed was enhanced from a low to a high level because of the combined effect of lowest deformability and shortest time scale for blade-induced deformations.

## 4. Conclusions

This study reports on the development of a sugar-based system as a model of a viscoelastic solid that can be used for the investigation of the cutting and fracture behavior at different rates. The model systems were designed as caramels, which represent a type of food still posing challenges regarding industrial cutting due to the pronounced dependency of the material response on cutting speed. Two major formulation-based factors known to influence mechanical properties, namely moisture content and solid fat content, and their effect on cutting behavior at different cutting speeds were investigated. Preliminary experiments had to be carried out to ensure the reproducibility of caramel cooking on a laboratory scale and the storage stability of finished caramels. Cutting tests displayed a transition from ductile to brittle behavior when cutting speed was increased. Viscous energy dissipation becomes less pronounced, making way for primarily brittle effects, i.e., anticipatory crack propagation. An analogy could be confirmed for decreasing moisture content and increasing SFC when speed is held constant. In all cases, it was found that, over the course of the transition, both maximum cutting force and cutting energy increase until increasing deformation energy, caused by reduced viscous effects, is counteracted by a decrease in fracture energy. From this point onwards, a decrease in these two parameters can be observed.

The developed reference material and the findings of this study lay the foundation for further investigations focusing on fracture behavior during cutting of viscoelastic foods, especially tensile stresses in the material. Parameters related to these aspects have to be identified for the numerical modeling of material fracture behavior in cutting processes, which will be the subject of further research.

## Figures and Tables

**Table 1:** Results from storage experiments. Water activity *a_W_*, Young’s modulus *E*, glass transition onset, *T_G,on_*, and endpoint temperature, *T_G,end_,* and change in specific heat capacity Δ*c_p_* measured over four days in 24 h increments. Mean values with different superscripts (a, b, ab) within a parameter and sample indicate statistical difference (*p* < 0.05).

Day	*a_W_*^1^ (−)	*E*^2^ (MPa)	*T_G,on_*^3^ (°C)	*T_G,end_*^3^ (°C)	Δ*c_p_* ^3^ (J/g·K)
Ref_120C_1, moisture ^1^: 7.64 ± 0.08%					
1	0.444 ± 0.003 ^a^	n.d.	−5.95 ± 0.09 ^a^	6.73 ± 0.09 ^a^	0.43 ± 0.02 ^a^
2	0.447 ± 0.001 ^a^	n.d.	−5.93 ± 0.23 ^a^	6.76 ± 0.08 ^a^	0.43 ± 0.02 ^a^
3	0.448 ± 0.004 ^a^	n.d.	−5.71 ± 0.62 ^a^	6.61 ± 0.36 ^a^	0.41 ± 0.02 ^a^
4	0.450 ± 0.003 ^a^	n.d.	−5.72 ± 0.42 ^a^	6.23 ± 0.04 ^a^	0.39 ± 0.01 ^a^
Ref_120C_2, moisture ^1^: 7.67 ± 0.08%					
1	0.445 ± 0.003 ^a^	8.75 ± 0.69 ^a^	−6.06 ± 0.12 ^b^	6.38 ± 0.16 ^a^	0.45 ± 0.02 ^a^
2	0.446 ± 0.001 ^ab^	7.51 ± 0.47 ^a^	−5.47 ± 0.10 ^a^	6.96 ± 0.06 ^a^	0.42 ± 0.01 ^a^
3	0.447 ± 0.002 ^ab^	8.33 ± 0.43 ^a^	−6.23 ± 0.10 ^b^	6.44 ± 0.00 ^a^	0.46 ± 0.01 ^a^
4	0.450 ± 0.001 ^b^	8.72 ± 0.72 ^a^	−5.62 ± 0.14 ^a^	6.45 ± 0.32 ^a^	0.42 ± 0.02 ^a^
Ref_115C_1, moisture ^1^: 9.72 ± 0.11%					
1	0.526 ± 0.003 ^b^	1.51 ± 0.29 ^a^	−15.46 ± 0.40 ^a^	−1.04 ± 0.72 ^a^	0.52 ± 0.01 ^a^
2	0.529 ± 0.002 ^b^	1.43 ± 0.21 ^a^	−14.97 ± 0.62 ^a^	−1.59 ± 0.58 ^a^	0.51 ± 0.02 ^a^
3	0.521 ± 0.002 ^a^	2.09 ± 0.44 ^b^	−15.35 ± 0.27 ^a^	−1.65 ± 0.15 ^a^	0.52 ± 0.02 ^a^
4	0.524 ± 0.002 ^ab^	2.15 ± 0.12 ^b^	−15.57 ± 0.02 ^a^	−1.86 ± 0.49 ^a^	0.50 ± 0.01 ^a^
Ref_115C_2, moisture ^1^: 10.42 ± 0.11%					
1	0.544 ± 0.004 ^a^	0.91 ± 0.18 ^a^	−17.50 ± 0.23 ^a^	−2.95 ± 0.23 ^a^	0.52 ± 0.01 ^a^
2	0.545 ± 0.002 ^a^	0.87 ± 0.13 ^a^	−17.95 ± 0.73 ^a^	−3.26 ± 0.73 ^a^	0.52 ± 0.02 ^a^
3	0.542 ± 0.002 ^a^	0.92 ± 0.13 ^a^	−17.28 ± 0.61 ^a^	−2.28 ± 0.61 ^a^	0.52 ± 0.00 ^a^
4	0.544 ± 0.002 ^a^	1.02 ± 0.21 ^a^	−16.98 ± 0.30 ^a^	−2.27 ± 0.30 ^a^	0.50 ± 0.01 ^a^

^1^ Data is arithmetic mean ± standard deviation (*n* = 3); ^2^ Data is arithmetic mean ± standard deviation (*n* = 7); ^3^ Data is arithmetic mean ± half deviation range (*n* = 2); n.d., not detected, due to technical issues.

## Data Availability

The data presented in this study are available on request from the corresponding author.

## Supplementary Materials

The following are available online at https://www.mdpi.com/article/10.3390/ma14143798/s1, Video S1: Cutting of a caramel sample (Ref, 7.98% moisture) at a speed of 400 mm/min (real time: 3.86 s), Video S2: Cutting of a caramel sample (Ref, 7.98% moisture) at a speed of 1000 mm/min (real time: 1.73 s), Video S3: Cutting of a caramel sample (Ref, 7.98% moisture) at a cutting speed of 2500 mm/min (real time: 0.76 s). All videos were recorded in slow motion at a frame rate of 500 frames per second.
